# Anaplastic transformation of thyroid cancer in mesentery metastases presenting as intestinal perforation: a case report

**DOI:** 10.1186/s40792-020-00959-x

**Published:** 2020-08-03

**Authors:** Kiyotaka Hosoda, Kei Kusama, Naoe Yanagisawa, Taiichi Machida, Akihito Nishio, Shinji Nakata, Ichiro Ito, Masahide Watanabe, Harutsugu Sodeyama

**Affiliations:** 1 Department of Surgery, Japanese Red Cross Society Nagano Hospital, 5-22-1 Wakasato, Nagano, Nagano 380-8582 Japan; 2 Department of Pathology, Japanese Red Cross Society Nagano Hospital, Nagano, Japan

**Keywords:** Anaplastic transformation, Peritoneal metastasis, Thyroid cancer, Intestinal perforation

## Abstract

**Background:**

Anaplastic thyroid carcinoma is a highly aggressive form of thyroid cancer associated with a very poor prognosis. Anaplastic transformation most commonly occurs in the thyroid itself or within regional lymph nodes. Here we report the case of a patient with papillary thyroid cancer, presenting with colon perforation as a result of anaplastic transformation of metastases in the mesentery tissue. There have been no previous reports of this form of anaplastic transformation.

**Case presentation:**

A 74-year-old man was admitted to our hospital, presenting with abdominal pain that he had been experiencing for 1 week prior to admission. The patient had a history of papillary thyroid carcinoma, for which he underwent a total thyroidectomy and mediastinal lymph node dissection 6 years earlier, and subsequently received radioactive iodine therapy for postoperative recurrence in the lung 2 years later. During the present reported admission, a computed tomography scan revealed a large intra-abdominal mass infiltrating into the colon and retroperitoneum and also highlighted the pneumoperitoneum. The patient was diagnosed with generalized peritonitis as a result of colon perforation, as such, we conducted an emergency laparotomy. Intraoperative findings showed a mass affecting the ascending colon and kidney, following which, an ileostomy and biopsy were completed. Poorly differentiated spindle cells were identified in the biopsy specimens, and histopathological and immunohistochemical findings revealed the absence of thyroid carcinoma cells. The tumor was therefore believed to be a primary sarcoma. Following surgery, the patient recovered from sepsis that had arisen as a result of colon perforation, however, rapidly developed systemic metastases and died 1 month post-operation. An autopsy was performed, and the patient was diagnosed with anaplastic papillary thyroid cancer at the mesentery site of metastasis. This conclusion was reached owing to the presence of the squamous differentiation of lymph node cells, and because tumor cells were positive results for paired-box gene 8 expressions.

**Conclusions:**

Anaplastic transformation of papillary thyroid carcinoma should be considered in the diagnosis of a large mesentery mass in patients with a history of papillary carcinoma. An appropriate biopsy and paired-box gene 8 immunostaining can be useful in confirming such a diagnosis.

## Background

Papillary thyroid carcinoma is typically a slow-progressing cancer with high rates of recovery. Anaplastic transformation of these tumors, however, forms a highly aggressive variant of thyroid cancer, associated with a very poor prognosis [1]. Anaplastic transformation most commonly occurs in the thyroid itself or within regional lymph nodes, and in rare cases, developing in distant metastases [2]. No reports have demonstrated anaplastic transformation from mesentery metastasis previously. Here we report the case of a patient with papillary thyroid cancer, presenting with colon perforation as a result of anaplastic transformation in mesentery metastases.

## Case presentation

**Fig. 1:**
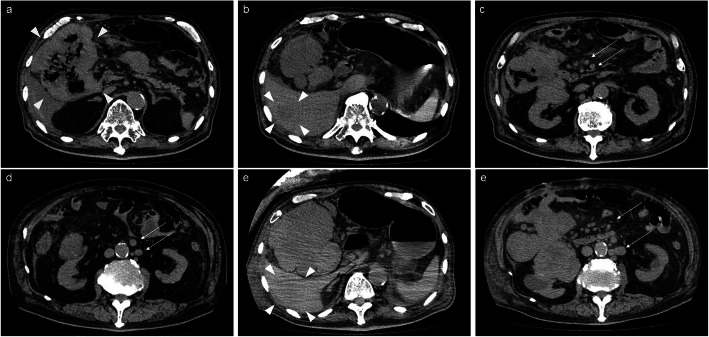
Computed tomography scan. Computed tomography shows a large intra-abdominal mass infiltrating into the colon and retroperitoneum (**a**, arrowhead) as well as the low-density area of the liver (**b**, arrowhead), peritoneal nodule (**c**, arrow), and lymphadenopathy in several paraaortic lymph nodes, suggesting metastases or dissemination (**d**, arrow). Pneumoperitoneum was visible, suggesting colon perforation near the tumor. Liver tumor, metastatic lymph node, and peritoneal dissemination were enlarged after 1 month (**e**, **f**, arrow, arrowhead)

A 74-year-old man was admitted to our hospital presenting with worsening abdominal pain he had been experiencing for 1 week prior to admission. The patient had a history of papillary thyroid carcinoma, pT4aN1bM0 stage I, for which he underwent a total thyroidectomy and mediastinal lymph node dissection 6 years earlier, and subsequently received radioactive iodine therapy for postoperative recurrence in the lung 2 years after the initial surgery. Upon admission, the patient’s laboratory data revealed renal dysfunction (blood urea nitrogen: 36.3 mg/dL and creatinine: 2.99 mg/dL) and an elevated white blood cell count and C-reactive protein levels (191,600/μL and 30.78 mg/dL, respectively). A computed tomography (CT) scan showed a large intra-abdominal mass infiltrating into the colon and retroperitoneum (Fig. 1a) as well as the low-density area of the liver (Fig. 1b), peritoneal nodule (Fig. 1c), and lymphadenopathy in several paraaortic lymph nodes suggesting metastases or dissemination (Fig. 1d). Pneumoperitoneum was visible from the CT scan, suggesting colon perforation close to the tumor. The patient was diagnosed with generalized peritonitis as a result of colon perforation, as such, we conducted an emergency laparotomy. Intraoperative findings showed that the mass had invaded the ascending colon and right kidney. Due to the extensive spread of the mass, the single-stage operation was difficult to perform, and so a protective ileostomy was performed, and a biopsy was obtained. Histology of the biopsy identified poorly differentiated spindle cells, and histopathological and immunohistochemical findings revealed the absence of thyroid carcinoma cells in the specimen. The tumor was therefore believed to be a primary intraperitoneal or retroperitoneal undifferentiated sarcoma.

Following surgery, the patient recovered from sepsis that had arisen as a result of colon perforation and was indicated to undergo chemotherapy for sarcoma. However, systemic metastases developed rapidly (Fig. 1e, f) and his general condition deteriorated soon after recovering from sepsis. The patients died without any chemotherapy 1-month post-operation.

**Fig. 2:**
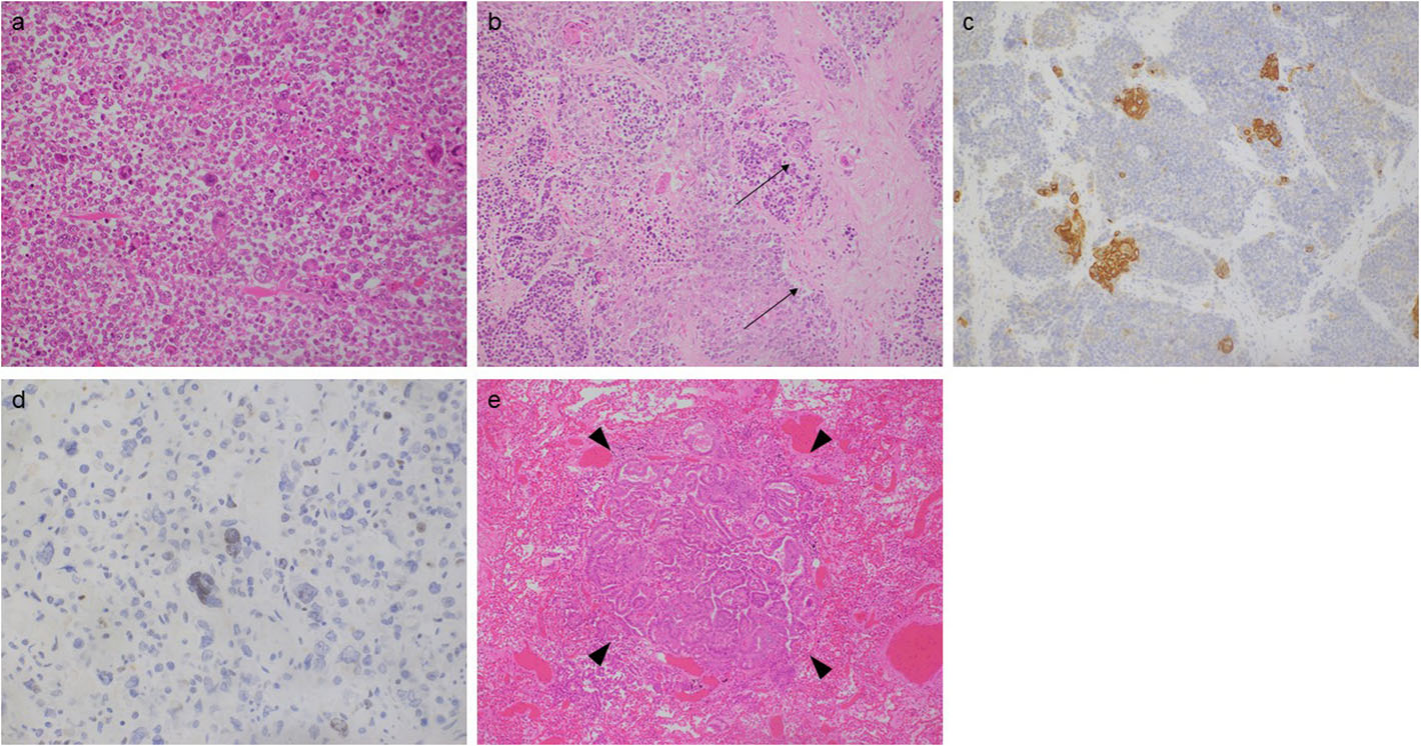
Pathological findings of the autopsy samples. **a** Tumor composed of pleomorphic spindle cells with eosinophilic cytoplasm and polymorphonuclear cell infiltration. (Hematoxylin and eosin stain, × 20). **b** Tumor cells with squamous differentiation (arrow) in the regional lymph node. (Hematoxylin and eosin stain, × 20). **c** Cytokeratin AE1/AE3-positive lesion shows squamous differentiation (× 20). **d** PAX-8 sporadically positive in the atypical lesion (× 40). **e** Tumors in the lung reveal morphology of typical papillary thyroid carcinoma (arrowhead). (Hematoxylin and eosin stain, × 10)

An autopsy was performed and identified the presence of tumors in the patient’s mesentery tissue, brain, lungs, paraaortic lymph nodes, liver, small intestine, and bone marrow. In the tumor located in the mesentery, necrotic activity was identified; the tumor was grayish-white in color, measured 18 × 15 × 10 cm, and had invaded the ascending colon, forming a cavity in the tumor. The tumor was composed of pleomorphic spindle cells with eosinophilic cytoplasm and polymorphonuclear cell infiltration (Fig. 2a), and did not contain lymph node components. It is notable that squamous differentiation was identified in tumor cells of regional lymph nodes (Fig. 2b). Immunohistochemical staining for markers showed the presence of p16, CD56, chromogranin A, and synaptophysin and absence of p63, CD 10, caldesmon, transcription factor 1 (TTF-1), and thyroglobulin (Tg). These atypical mesenteric lesions also stained positive for cytokeratin AE1/AE3, exhibited squamous differentiation (Fig. 2c), and was sporadically positive for paired-box gene 8 (PAX-8) (Fig. 2d). MDM2 expression was not detected by fluorescence in situ hybridization. These findings suggest that this tumor was an undifferentiated carcinoma rather than a sarcoma. In addition, the tumors found in the lungs and brain had morphology that is typical of papillary thyroid carcinoma (Fig. 2e), with no other malignancies found. Based on these findings, the patient was diagnosed with anaplastic papillary thyroid cancer at a site of metastasis in the soft tissue of the mesentery.

## Discussion

Papillary thyroid carcinoma is the most common type of malignant thyroid tumor, which typically progresses slowly and has an excellent prognosis [3]. While anaplastic transformation occurs in only 1–2% of malignant thyroid tumors, patients with these cancers have a dismal prognosis, with a median survival time of < 6 months [4, 5]. In anaplastic thyroid cancer patients with distant metastases (stage IVC), chemotherapy is the only treatment approach available. In chemotherapy regimens including doxorubicin or paclitaxel, over 50% of patients responded to the treatment; however, complete response was only observed in a few of these patients [6, 7]. The efficacy of molecular targeted drugs such as lenvatinib, a multi-tyrosine kinase inhibitor, has also been reported in recent years [8].

Anaplastic transformation most commonly occurs in the thyroid itself or within regional lymph nodes [9] and rarely develops at distant metastasis sites. Only 14 such cases were reported between 2000 and 2019; the characteristics of these cases are summarized in Table 1. Of these 14 patients, six were men (42.9%), eight were women (57.1%), and the median age of patients was 63.5 (range: 52–83) years. The time between primary diagnosis and anaplastic transformation ranged from 7–49 years. All patients died within 24 weeks (range: 2–24 weeks) of anaplastic transformation detection. The sites of anaplastic transformation included the submandibular region [10], liver [11], breast [12], retroperitoneum [13, 14], lung [2, 15, 16], shoulder [17], pelvis [18], mandible [19], and pleura [20, 21]; there have been no previous reports of anaplastic transformation occurring in the mesentery tissue. Furthermore, reports of mesentery metastases are rare in both papillary thyroid cancer and more common forms of anaplastic thyroid cancer [22, 23], making the patient’s diagnosis in this case study an extremely rare and novel case.

**Table 1:** Previous reports of anaplastic transformation of thyroid carcinoma developed from distant metastasis sites

Author	Year	Age	Sex	Location	Duration from primary diagnosis (years)	Immunostaining			^131^I therapy	Survival (weeks)
						TTF-1	Tg	PAX-8		
Sumida et al.	2000	60	Female	Submandibular region	7	N/A	N/A	N/A	None	20
Takeshita et al.	2008	81	Female	Liver	3	Negative	Focally	N/A	Yes	16
Angeles-Angeles et al.	2009	58	Female	Breast	20	Focally	Focally	N/A	Yes	N/A
Al-Qsous et al.	2010	83	Male	Lung	10	N/A	Weak	N/A	None	N/A
Sotome et al.	2010	82	Female	Retroperitoneum	18	Negative	Negative	N/A	Yes	2
Kaushal et al.	2011	52	Female	Shoulder	8	Positive	N/A	N/A	Yes	8
Nakayama et al.	2012	55	Female	Pelvis	12	Negative	Negative	N/A	Yes	14
Abe et al.	2014	61	Male	Lung	10	Positive	Negative	N/A	Yes	24
Solomon et al.	2015	64	Male	Retroperitoneum	28	Weak	Negative	Positive	Yes	3
Ambelil et al.	2016	76	Female	Mandible	7	Negative	Negative	Positive	Yes	N/A
Kim et al.	2017	61	Female	Pleura	19	Positive	Negative	Positive	None	16
Okuyama et al.	2017	63	Male	Lung	49	N/A	N/A	N/A	Yes	20
Capron et al.	2019	77	Male	Pleura	9	Negative	Negative	Positive	Yes	N/A
Present case	2020	77	Male	Mesentery	6	Negative	Negative	Positive	Yes	4

*N/A* not applicable

In recent years, some reports have suggested that ^131^I therapy, which is often administered to patients with recurrent papillary thyroid carcinoma, particularly in those with limiting tumor recurrence, can be associated with anaplastic transformation of papillary thyroid carcinoma [11, 24, 25]. In those reports, patients with anaplastic carcinomas were thought to have DNA damage induced by ^131^I therapy and the combination of p53, a representative tumor suppressor gene, mutations, and DNA damage induced by ^131^I therapy is reported to lead to anaplastic transformation [25]. Of the 14 patients reported to have had anaplastic transformation at distant metastatic sites, 11 patients, including the patient in this case, had received ^131^I therapy. Though this evidence is insufficient to suggest a causal relationship, these findings are suggestive of a correlation between ^131^I therapy and anaplastic transformation.

In this case study, we considered the possibility of an anaplastic papillary carcinoma owing to the patient’s history of recurrent papillary thyroid carcinoma. However, we could not confirm a diagnosis of anaplastic thyroid carcinoma at the time of operation, owing to histopathological and immunohistochemical findings indicating the absence of thyroid carcinoma markers in the tumor. The diagnosis of anaplastic papillary thyroid cancer could only be made with the detection of PAX-8 expression in a highly atypical lesion of the tumor located in the mesentery tissue as well as with the finding of the squamous differentiation of metastatic lymph node cells.

PAX-8 is a transcription factor involved in the process of cell differentiation in the thyroid, kidneys, and Müllerian duct. It is often expressed in thyroid cells even if an anaplastic transformation has occurred [26], whereas the expression of other thyroid-specific markers such as TTF-1 and Tg is often lost after anaplastic transformation [27]. This is also true for anaplastic transformation occurring in distant metastases. PAX-8 immunostaining is therefore valuable in the diagnosis of anaplastic thyroid cancers. In this case, we did not check PAX-8 expression because we thought that it will not contribute to the diagnosis since PAX-8 expression can be detected in different tumors other than thyroid tumor, such as renal cancer. In hindsight, we should have checked PAX-8 immunostaining, as it would have led to the correct diagnosis.

However, it should be noted that such a disease is difficult to diagnose based only on the expression of PAX-8, and it is important to rule out other cancer types by histological examination or image-based findings. Moreover, in some cases, diagnoses are difficult to obtain, since biopsy specimens from several sites are requiring for histological examination or immunostaining before an accurate diagnosis can be confirmed. Obtaining biopsy samples from lymph nodes or other sites of metastasis can therefore be also helpful in diagnosing cases of anaplastic transformation.

## Conclusions

Anaplastic transformation of papillary thyroid carcinoma can occur in mesentery sites of metastases. This disease should be considered in the diagnosis of a large mesentery mass in patients with a history of papillary carcinoma. The histological examination and PAX-8 immunostaining of multiple biopsies from the tumor or lymph nodes may be useful in the confirmation of this diagnosis.

## Data Availability

All data generated during this study are included in this published article.

## Abbreviations

CT: Computed tomography
TTF-1: Transcription factor 1
Tg: Thyrogloblin
PAX-8: Paired-box gene 8
